# Persistent or Recurrent Urinary Incontinence 8 Years After Midurethral Sling Surgery: A Retrospective Cohort Study

**DOI:** 10.1007/s00192-025-06183-1

**Published:** 2025-07-07

**Authors:** Bianca B. Mengerink, Emma E. Pluymen, Sanne A. L. van Leijsen, Esmee N. de Jong, John P. F. A. Heesakkers, Kirsten B. Kluivers

**Affiliations:** 1 Department of Obstetrics & Gynecology, Bernhoven Hospital, Nistelrodeseweg 10, 5406 PT UDEN, The Netherlands; 2 Department of Obstetrics & Gynecology, Radboud University Medical Center, Nijmegen, The Netherlands; 3 Department of Obstetrics & Gynecology, Rijnstate Hospital, Arnhem, The Netherlands; 4 Department of Obstetrics & Gynecology, Maxima Medical Centre, Veldhoven, The Netherlands; 5 Department of Urology, Maastricht University Medical Centre, Maastricht, The Netherlands

**Keywords:** Urinary incontinence, Stress urinary incontinence, Midurethral sling surgery, Recurrent urinary incontinence, Persistent urinary incontinence

## Abstract

**Introduction and Hypothesis:**

Midurethral sling (MUS) surgery improves the quality of life of women with stress urinary incontinence (SUI). Nonetheless, treatment can fail leaving some women who still suffer from urinary incontinence post-surgery. This study determines the prevalence of persistent or recurrent urinary incontinence 8 years after MUS surgery and describes the type and effectiveness of additional treatments.

**Methods:**

This retrospective cohort study is a long-term follow-up study on women who participated in a randomized controlled trial (RCT) on the value of urodynamics and who underwent MUS surgery (VUSIS-2 study). Data were collected through medical file review in 12 of 30 recruiting hospitals, representing 68.2% of inclusions. Data on postoperative symptoms, additional diagnostics and treatments were collected.

**Results:**

Of 578 VUSIS-2 participants, 301 (52.1%) medical files were analysed. Urinary incontinence symptoms were reported in 71 cases (23.6% [95% CI 19.1–28.7]) over a median follow-up of 7.8 years. SUI symptoms were reported in 38 patients (12.6%), including 18 patients (6.0%) [95% CI 2.6–9.3]) with persistent and 20 patients (6.6%) [95% CI 3.1–10.1] with recurrent SUI. Urgency urinary incontinence was reported in 51 medical files (16.9%) [95% CI 11.9–21.6], whereof 18 patients had mixed urinary incontinence complaints. Additional treatment was received in 42 patients (59.2% of incontinent cases [95% CI 47.5–69.8]), of whom nine (3.0%) received additional surgery (3 excisions/removals (1.0%) and two additional MUS placements (0.7%)).

**Conclusions:**

In this retrospective long-term follow-up study among women receiving MUS surgery for predominant SUI, postoperative urinary incontinence symptoms were reported in nearly a quarter of cases. One-third of these women did not receive additional treatment. The prevalence of repeat surgery was 5.6%.

## Introduction

Stress urinary incontinence (SUI) is defined as involuntary loss of urine on effort, coughing, sneezing or physical exertion. It is a common problem among adult women with an estimated prevalence of 25% to 57% [1, 2]. SUI is a benign condition but the symptoms have a great impact on social, physical and psychological well-being, associated with low self-esteem and social isolation [3].

Midurethral slings (MUS) were introduced in the mid 1990s. It has been demonstrated that MUS surgery is more effective in women with moderate and severe SUI as compared to pelvic floor physiotherapy [4] and colposuspension [5]. Moreover, MUS has a lower perioperative morbidity than colposuspension [5–7]. In some countries, however, the use of synthetic MUS has been restricted because of the potential risks. As a consequence of the public concern on the use of synthetic mesh, the NICE guideline, amongst others, do not recommend MUS procedures anymore as first choice treatment for urinary incontinence [8].

MUS surgery has a positive impact on the quality of life of women with SUI [9], but not all women have a benefit. Persistence or recurrence of urinary incontinence (UI) is a challenging problem. The decision for and the outcome of further treatment depends on multiple factors such as the type of UI, additional pelvic floor symptoms, type of the first procedure, time till failure and patient factors such as BMI, comorbidities and patients’ attitude towards further treatment/surgery [10]. Long-term follow-up studies (10–17 years) have shown objective cure rates ranging from 84 to 91% and subjective cure rates from 57 to 77% [6, 11–13]. Despite these high success rates, these rates imply that there is a significant number of patients treated with MUS who experience symptoms of persistent or recurrent SUI after MUS surgery. Repeat surgery rates for persistence or recurrence of SUI varies in the available studies: between 5.2% and 17% during follow-up up till 10 years [10, 11, 14–17].

The aim of this retrospective cohort study was to evaluate the clinical management of women who experienced symptoms and signs of UI up to 10 years after MUS surgery. A distinction between persistent or recurrent SUI and further types of UI was made, and the additional diagnostics and treatments and their outcomes are reported.

## Materials and Methods

This report describes a retrospective cohort study of the follow-up of women who participated in the VUSIS-II study [18]. A retrospective medical file review at 6 to 10 years after MUS placement was performed. We assessed the medical files for reported postoperative lower urinary tract symptoms (LUTS), and additional diagnostics and treatments.

The VUSIS-II study was an RCT regarding the value of urodynamics prior to midurethral sling surgery. It assessed the outcomes of treatment in 578 women with predominant SUI in 6 academic and 24 nonacademic hospitals in the Netherlands from January 2009 through November 2010. Women were eligible in case conservative treatment had failed, and they had a clinical indication for surgical SUI treatment. SUI had to be demonstrated by a positive cough stress test and/or an incontinence episode recorded on a bladder diary. Patients were excluded in case of prior incontinence surgery, pelvic organ prolapse of Pelvic Organ Prolapse Quantification (POP-Q) stage > 2, or post void residual bladder volume of 150 mL or more (on ultrasonography or catheterization). All women in the study underwent urodynamics. Urodynamics were called discordant when the results did not confirm the history of SUI or showed relative contraindications for surgical SUI treatment: stress incontinence was not demonstrable, detrusor overactivity, hypo contractility of the bladder, poor flow, residual urine, outflow obstruction, small cystometric capacity, raised sensibility of the bladder, lowered sensibility of the bladder, low level of compliance. In case of discordant findings, women (*n* = 126, 22%) were randomized in the VUSIS-II study between immediate MUS surgery or individually tailored treatment, which could include MUS surgery. Four hundred fifty-two patients (78%) had concordant findings and were not randomized. Their outcome was prospectively followed for 1 year and reported as results of the VUSIS-II study [19]. The choice for the type of MUS surgery (transobturator or retropubic) was left to the discretion of the surgeon, but single incision sling was not allowed.

In the present retrospective study, participants from 12 out of 30 centres who had undergone MUS surgery as initial treatment in the VUSIS-II study were included. We contacted all 30 participating hospitals for retrospective medical file review. The other 18 centres did not respond despite reminders, these were mainly the centres with low numbers of study participants. Pre- and postoperative data were extracted from the VUSIS database and all data concerning the long-term follow-up to 10 years after primary surgery were extracted from the medical files at the gynaecology and urology departments. In the Netherlands, women can return to their medical specialist within 1 year after their last hospital visit without a new referral. In case medical files were missing or no documentation was available after surgery, women wereexcluded from analysis.

The primary outcome of this study was the reported presence and type of UI symptoms in the medical file. The women were not contacted and there was no standard follow-up visit planned for this study. As secondary outcome, data on further diagnostics and treatments for lower urinary tract symptoms and tape complications as well as outcomes in case of additional treatment were collected.

Persistent UI was defined as UI symptoms after MUS surgery documented in the medical files at regular follow-up visits after surgery. We defined recurrent UI as the renewed onset of UI as derived from the medical files in women who reported no postoperative UI until 12 weeks after surgery. Subjective cure was defined as the absence of UI as derived from the medical files.

Institutional review board approval was obtained for this follow-up study (file number 2017–3358 CMO region Arnhem/Nijmegen). The original VUSIS-II was funded through ZonMw, the Dutch Organization for Health, Research and Development, projectnumber 945–07–203. There was no funding for this follow-up study.

All analyses were performed using SPSS 24. Baseline characteristics were summarized using descriptive statistics. *T*-test for continual data and Pearson chi-square test for nominal data were used to compare groups. Cumulative incidence was estimated using the life table method. Univariate and multivariable analysis was used to assess for risk factors. Possible risk factors were determined to include age, BMI, parity, type of MUS surgery and preoperative complaints of urge urinary incontinence (UUI), variables with *p* values < 0.20 in univariate analysis were included in the multivariable logistic regression analysis. A backward stepwise likelihood ratio (LR) procedure was applied. *P* values < 0.05 were considered statistically significant. Signs and symptoms of UI and additional diagnostics and treatments are presented in descriptive tables or described as percentages (with 95% CI).

## Results

Out of the 578 women who participated in the VUSIS-II study, 506 had undergone MUS as initial treatment. Our study population consists of 301 (59.5%) inclusions from 12 different study sites, after excluding 44 women with insufficient data or where no follow-up visit was available during data collection. The mean number of participants in the VUSIS-II study per study site was 29.3 (range 2–117)) for the 12 included study sites and 10.5 (range 1–37) for the nonresponders.

Preoperative patient demographics of the study population and those of the participants from the nonresponding study sites are summarized in Table 1. Eighty-eight (29.3%) women had received a retropubic tension free vaginal tape (TVT) and 213 (70.7%) a trans obturator tape (TOT).

**Table 1:** patient demographics

	Inclusions *n* = 301 *mean (*± *SD)*		Participants from nonresponding study sites *n* = 205 *mean (*± *SD)*		*P* value
Age (years)	53.4 (11.6)		52.3 (12.2)		0.52
BMI	27.1 (4.8)		26.5 (5.1)		0.41
Parity	2.3 (1.1)		2.3 (1.0)		0.54
	*n*	*percentage*	*n*	*percentage*	
Type of UI before surgery:					
- Pure SUI	93	30.9%	77	37.6%	0.14
- MUI, predominant SUI	208	69.1%	128	62.4%	
Type of MUS:					
- Retropubic tape	88	29.3%	29	14.1%	< 0.01
- Transobturator tape	213	68.8%	122	59.5%	
- *unknown*			*54*	*26.3%*	
PGI-I 12 months after surgery					
- Better	242	80.1%	155	75.6%	0.32
- Same	14	4.7%	11	5.4%	
- Worse	9	3.0%	2	1.0%	
- *Missing*	*40*	*13.3%*	*37*	*18.0%*	
UDI-UI 12 months after surgery					
- No UI	190	63.1%	105	51.2%	0.06
- UI	83	27.5%	67	32.6%	
- *missing*	*28*	*9.3%*	*33*	*16.1%*	

*BMI* body mass index, *UI* urinary incontinence, *SUI* stress urinary incontinence, the definition of pure SUI was based on the absence of UUI in the questionnaire, *MUI* mixed urinary incontinence, *MUS* midurethral sling. *P* value is tested by *t*-test for continual data and by Pearson chi-square test for nominal data

The participants in our study population had a median age of 52.3 years at time of surgery (IQR 45–61 years). The median follow-up time was 7.8 years, which was the interval between surgery and data collection in case files. The median time between surgery and the most recent documented follow-up visit in the medical records was 2 months (range 0 to 84 months). It is important to note that while our follow-up study assessed symptoms at 8 years post-surgery, the most recent recorded contact in the patient files varied significantly, with some patients having only documented follow-up after 3 weeks and others having a recorded visit as late as 84 months after surgery.

### Postoperative UI and Other Complaints

**Fig. 1:**
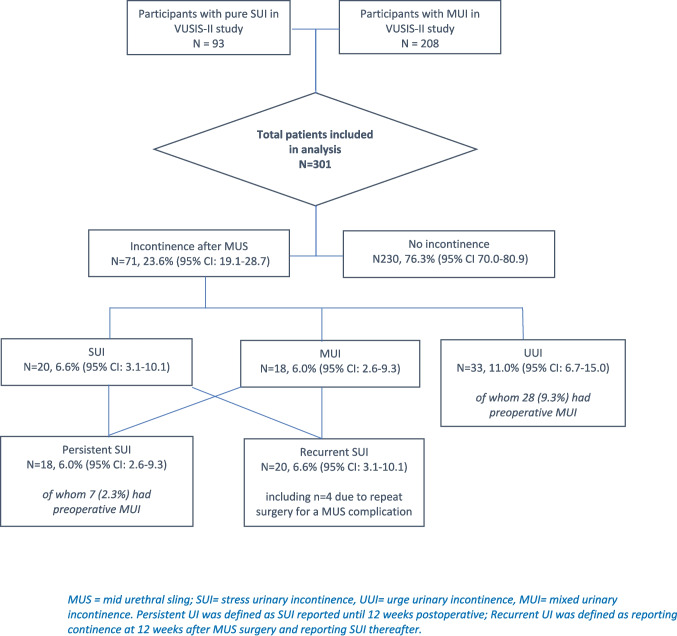
Urinary incontinence after surgery

**Fig. 2:**
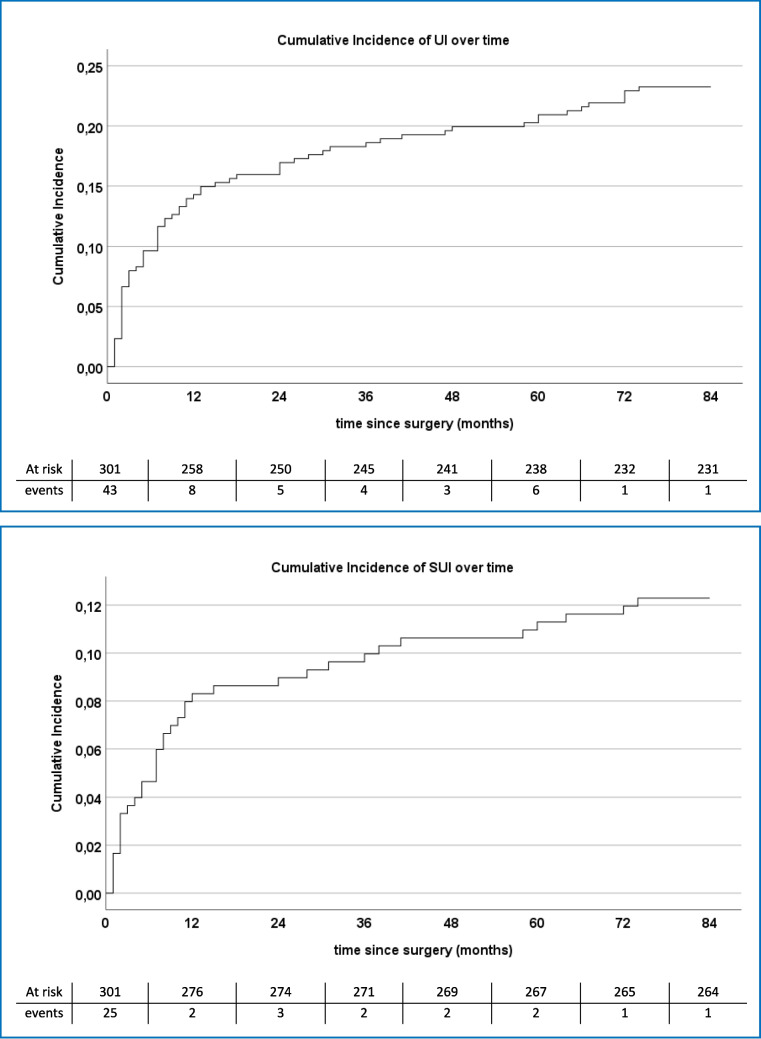
Cumulative incidence of Urinary Incontinence (UI) and Stress Urinary Incontinence (SUI) over time

In the medical files of 71 women (23.6%, (95% CI 19.1–28.7)) symptoms of postoperative UI were reported SUI in 20 (6.6%), UUI in 33 (11.0%) and MUI in 18 (6.0%). In 18 women (6.0%) persistent SUI was reported, whereas in 20 women (6.6%) recurrent SUI was reported (i.e. after initial continence after MUS surgery) (Fig. 1). In four out of these 20 patients this recurrent incontinence was due to surgery because of MUS complications. Among the patients experiencing pure UUI complaints after surgery, five (1.7%) patients complained of de novo UUI. Results are shown in a cumulative incidence curve (Fig. 2).

No associations of postoperative UI with age, BMI and parity were observed. The presence of UUI before MUS treatment is a risk factor of having postoperative UI. There was no correlation found between the type of MUS and postoperative UI at 8 years (Table 2).

**Table 2:** Possible risk factors for postoperative UI

Risk factor	*N*	Urinary incontinence *n* = 71 (%)		No urinary incontinence *n* = 230 (%)		Univariate analysis OR [95%CI]	Multivariable analysis OR [95%CI]
		*N*	(%)	*N*	(%)		
Age	301	52.5		53.7		0.99 [0.97–1.01]	–
BMI	255	27.7		27.0		1.03 [0.97–1.09]	–
Parity	288	2.5		2.2		1.21 [0.96–1.53]	1.18 [0.92–1.49]
Type of UI before surgery:							
pure SUI	93	15	(21.2)	78	(33.9)	Reference	Reference
MUI	208	56	(78.8)	152	(76.1)	*1.91 [1.02–3.60]*	*2.02 [1.04–3.94]*
Type of MUS:							
TVT	89	24	(33.8)	65	(28.3)	0.77 [0.43–1.36]	–
TOT	112	47	(66.2)	165	(71.7)	Reference	–

*BMI* body mass index, *SUI* stress urinary incontinence, *MUI* mixed urinary incontinence, *TVT* trans vaginal tape, *TOT* trans obturator tape, *n* number. Variables with *p* values < 0.20 in univariate analysis were included in the multivariable logistic regression analysis

A backward stepwise likelihood ratio (LR) procedure was applied. *P* values < 0.05 were considered statistically significant

Further complications reported were tape exposure (*n* = 7, 2.3%) and signs and symptoms of obstructive micturition, including urinary retention (*n* = 22, 7.3%). After 14 transections or excisions of MUS as re-interventions, seven women (50%) suffered from complaints of UI. In three women out these seven, there was recurrent UI after repeat surgery. Furthermore nocturia (*n* = 24, 8.0%), frequency (*n* = 17, 5.6%), dyspareunia (*n* = 9, 3.0%), (groin)pain (*n* = 13, 4.3%), recurrent urinary tract infection (*n* = 19, 6.3%) and various other minor symptoms (*n* = 6, 2.0%) were described.

### Urodynamics

During the follow-up period, urodynamics were repeated in 14 cases. Findings could be retrieved in nine cases. Appendix Table 3 shows an overview of the symptoms and the results from the preoperative and postoperative urodynamics.

**Table 3:** The pre- and postoperative urodynamic findings in patients in whom urodynamics were repeated postoperatively because of LUTS after MUS placement

Patient	1	2	3	4	5	6	7	8	9
Preoperative symptoms#	MUI	MUI	MUI	MUI	SUI	MUI	MUI	MUI	MUI
Year MUS	2010	2009	2010	2010	2009	2010	2009	2009	2010
Postoperative symptoms	MUI	UUI	UUI	MUI	No UI; UTI	UUI	UUI	MUI	MUI, obstructive micturation
Date postoperative urodynamics	2013	2010	Jan 2016	2013	2013	2010	2010	2017	2016
Cystometric capacity*	312/420	400/80	452/332	150/200	359/240	missing/100	308/254	466/353	116/50
Urodynamic SUI*	no/no	yes/missing	no/no	yes/no	no/no	no/missing	yes/no	No/missing	no/yes
Urodynamic DO*	no/no	yes/yes	no/no	no/no	no/yes	yes/yes	no/yes	no/no	no/yes
Residual urine (ml)*	no/no	No/no	no/no	47/no	11/no	missing/no	no/no	100/60	no/no
MUCP (mm H_2_O)*	25/missing	50/54	78/79	78/normal	80/missing	35/33	63/missing	68/40	103/missing
Transmission (%)*	32/missing	31/91	70/76	70/normal	79/missing	91/99	18/missing	83/70	71/missing
Additional therapy	PTNS		Medication	PTNS, medication	Antibiotics	Medication	Botulin inj	Medication	Transection tape

*LUTS* lower urinary tract symptoms, *MUS* midurethral sling, *SUI* stress urinary incontinence, *MUI* mixed urinary incontinence, *DO* detrusor overactivity, *MUCP* maximal urethral closure pressure, *PTNS* percutaneous tibial nerve stimulation

*preoperative/postoperative findings on urodynamics

#note that only patients with ‘MUI predominant SUI’ were included in the VUSIS-II study in case of MUI symptoms

### Additional Treatment

Among the 71 women, 23.6% (95% CI 19.1–28.7) who reported postoperative UI, 42 women, 59.2% (95% CI 47.5–69.8) of incontinent cases, received at least one type of additional treatment. Treatments received were medication (*n* = 33, 46.5% of women receiving treatment), pelvic floor physiotherapy (*n* = 18, 25.4%), repeat MUS surgery (*n* = 5, 7.0%), bulking agent injections (*n* = 2 2.8%), percutaneous tibial nerve stimulation (PTNS) (*n* = 1, 1.4%) and botulinum toxin (*n* = 1, 1.4%)). Medical treatment for UUI mostly consisted of antimuscarinics (*n* = 17, 51.5%). Other medications subscribed were antibiotics and vaginal estriol.

For the group of women with persistent or recurrent SUI symptoms after initial MUS surgery (*n* = 38, 12.6% (95% CI 8.1–16.9)), additional treatment was performed in 24 women, 33.8% (95%CI 18.8–45.4) of incontinent cases.

There were five additional MUS placements, regarding a reoperation rate for SUI of 1.7%. Repeat retropubic tapes were performed twice because of persistent pure SUI or MUI symptoms. The other three repeat (retropubic *n* = 2, MiniArc *n* = 1) tapes were performed after tape removal, e.g. transection of the initial tape. In Appendix Table 3, an overview is given of all women receiving additional MUS surgery after initial MUS surgery.

In our study population, there were in total five excisions of tape exposure and nine transections of the tape. Tape revision had occurred in nine women with postoperative UI (12.9%) (see Appendix Table 4).

**Table 4:** Patients who suffered from urinary incontinence after initial MUS surgery and underwent repeat surgery

	Type of hospital	Type of pre- operative UI	Type of initial sling	Type of post- operative UI	Recurrent or persistent UI	Additional symptoms and treatment	Outcome surgery	Second additional treatment	Outcome additional treatment
Patient A	Nonacademic	MUI	TOT	UUI	–	Transection for obstructive micturition	Recurrent SUI	Bulkamid and vesicare	
Patient B	Nonacademic	MUI	TVT	UUI	–	Transection for obstructive micturition	Recurrent SUI	Repeat MUS: single-insicion sling	Dyspareunia, exposure single-insicion sling, resection and physiotherapy
Patient C	Academic	MUI	TOT	SUI	Recurrent	Transection for obstructive micturition	Recurrent SUI	Bulkamid and repeat MUS: retropubic tape	Antibiotics for Recurrent UTIs
Patient D	Academic	SUI	TOT	UUI	Persistent	Transection	Persistent UUI	Mirabegron	Unknown
Patient E	Academic	MUI	TVT	MUI	Recurrent	Tape removal for exposure	Persistent MUI	Second tape removal, vesicare, mirabegron, botuline injections	Persistent MUI
Patient F	Academic	MUI	TOT	SUI	Recurrent	Tape removal for exposure	Persistent SUI	Physiotherapy	Cured
Patient G	Academic	MUI	TOT	MUI	Recurrent	Repeat MUS: retropubic tape	Cured	Tape removal for exposure and second repeat MUS: retropubic tape	Unknown
Patient H	Academic	MUI	TOT	SUI	Recurrent	Tape removal for exposure	SUI	Repeat MUS: retropubic tape	Cured
Patient I	Academic	MUI	TOT	SUI	Recurrent	Repeat MUS: retropubic tape	Cured		

*UI* urinary incontinence, *MUI* mixed urinary incontinence, *SUI* stress urinary incontinence, *UUI* urge urinary incontinence, *TOT* trans obturator tape, *TVT* trans vaginal tape, *UTI* urinary tract infection

### Outcome of Additional Treatment

The outcome of repeat MUS surgery showed that all four women on whom information was available became continent, whereas no information could be found on a fifth case. The outcome of women who received physiotherapy or medication was poorly documented so no firm conclusions can be made.

## Discussion

In the present study, we retrospectively evaluated the long-term follow-up in a large cohort of women who had MUS in the VUSIS-II study. We summarize the symptoms and treatments as reported in the medical files. The women may experience persistent, recurrent or de novo symptoms of UI (SUI, UUI and MUI) and had additional diagnostics and treatments up to 10 years after their initial MUS surgery. The overall rate of incontinence after initial MUS surgery was 23.6% (95% CI 19.1–28.7) in our population, of whom 59.2% (95% CI 47.5–69.8) received additional treatment. The most common type of postoperative UI in this group of women receiving MUS for predominant SUI was postoperative UUI. As expected, having MUI predicted the occurrence of postoperative UI. The overall re-treatment rate was thereby 14% in a study population of 301 patients with a median follow-up of 7.8 years (range 6–10 years) after MUS. In total, there were 17 sling-related repeat surgeries done, whereof in five women (1.7%) a repeat MUS was performed.

As we did not have all the medical (outcome-) data on the effectiveness of the additional treatments, it is not possible to draw reliable conclusions on the overall effectiveness of the various types of additional treatments. What we can conclude, however, is that a transobturator route sling was most often followed by a retropubic sling and was successful in at least four out of five patients who were cured from UI after repeat surgery.

In our study, having preoperative MUI was an independent risk factor for postoperative UI, meaning that a component of UUI before MUS is positively correlated with persistent complaints of UUI after MUS. It is important to note that in our cohort, all women with MUI presented with predominant SUI. The result is expected since MUS does not address urgency incontinence. In our study, we did not find a significant correlation for obesity, parity, age and type of sling for reported UI after MUS surgery.

A variety of factors have been suggested by other study groups to be associated with persistent or recurrent SUI after MUS procedures, including demographic factors such as advanced age, obesity, comorbidity, the presence of severe cystocele and the presence of preoperative mixed incontinence [20–22]. We were not able to confirm further factors.

De novo UUI was observed in 1.7% of all women who underwent MUS surgery, which is lower than the 5.6% prevalence reported by Berger et al. [15]. This discrepancy may be explained by the fact that women in our study only consulted their physician in case of complaints. In the literature, symptoms are often assessed via questionnaires, and the severity of de novo UUI is not typically reported. Consequently, women with little bother may have been counted in those studies.

In this study, in 76.3% of cases no complaints of UI were reported. Success of MUS is optimally evaluated by objective and subjective cure rates, which is either by exam or by patient-reported questionnaires. This study is based on the symptoms of UI reported in medical files. This difference in methodology makes direct comparison with other studies challenging. For context, in the 1-year VUSIS-2 follow-up data, 68.3% of women reported subjective cure defined as ‘(very) much improved’ on the PGI-I scale. In addition, long-term studies evaluating the effectiveness of MUS have reported subjective cure rates ranging from 57 to 77% [6, 11–13].

Various studies have reported a range of reoperation rates following MUS surgery. In the VUSIS-2 study, we observed a re-operation rate of 2.8% (14 out of 506 patients) within 1 year [18]. Long-term data from two large cohorts reported slightly higher reoperation rates compared to our findings. Dejene et al. reported a 7.9% risk of sling revision within 15-years, with nearly half of these revisions due to mesh exposure [16]. Berger et al. found an overall revision rate of 6.0% after 9 years [15]. In our current study the total re-operation rate after 8 years—including repeat MUS, excision exposure, transaction MUS, MUS removal—was 5.6% (*n* = 17). Notably, repeat MUS was performed in 1.6% (*n* = 5) of patients, which is in line with the literature [15]. While our reoperation rates appear slightly lower, this may reflect an underestimation of re-operations. It might be that patients underwent treatment in another hospital than the study site, following a referral by their general practitioner. However, normally the initial surgeon would be informed about this, leading to a report in the medical file.

Urodynamic test results have been described in Appendix Table 3. Owing to a lack of data, interpretation and reflection of the results and the heterogeneity of these data we cannot give reliable conclusions on these results.

Strengths of the study were our relatively large study population and long-term follow-up until 10 years; 301 women were included in this study which adds to the robustness of our results. The results from the PGI-I and UDI at 12-months follow-up were similar between the group of participants included in this study and of participants from nonresponding study sites, which supports the generalizability of our study population.

In addition, we did include women from different centres (academic and nonacademic), thereby broadening generalizability. Finally, our retrospective study was based on a prospective design, which provided us with reliable preoperative data.

Several limitations should be acknowledged. An important limitation of this study is the missing data from nonresponding study sites which may have led to selection bias. Our study population represented approximately 60% of the original VUSIS-2 study cohort. Analysis of the available demographic data indicated that this group was comparable to the nonresponder group in terms of baseline characteristics. However, we observed a higher proportion of patients who underwent a transobturator tape procedure within the nonresponder group. Despite this, previous studies have shown that subjective and objective cure rates for retropubic and transobturator approaches are comparable [9]. Furthermore, there was a trend suggesting lower rates of urinary incontinence in our study population, as indicated by the UDI-UI score at the 12-month follow-up. Consequently, the reported prevalence of urinary incontinence symptoms in our dataset may underestimate the true burden of urinary leakage within the total study population. A much larger population with standardized follow-up would be needed to draw firm conclusions on the optimal treatment after MUS failure. This may only be feasible for such a long-term follow-up in national registry studies which are not available in most countries. Another factor is that women may have moved/relocated, switched to another hospital or sought other types of treatment, such as a paramedic, physiotherapist, general practitioner and others. In that case, they would not have been captured in this study as an MUS failure.

It is also important to note that we defined the presence of incontinence based on the assessments of the women as captured in their medical files, next to the additional preoperative information from the VUSIS database. We expect that an underestimation of the incidence of incontinence after MUS is more likely than an overestimation. We had, however, no information available on the severity of the UI symptoms. Therefore, it could also be that relatively mild incontinence, preceded by severe preoperative incontinence, still led to a satisfactory result for that patient.

In conclusion, our study revealed that nearly one in four women experienced postoperative urinary incontinence within 8 years of undergoing midurethral sling surgery. Stress urinary incontinence (SUI) was identified as the most prevalent type of urinary incontinence. Additionally, one in six women reported urge urinary incontinence (UUI). Furthermore, one in seven women required further treatment due to persistent or recurrent symptoms following the procedure.

## Data Availability

The data are not publicly available due to privacy or ethical restrictions.
